# Broadband-Tunable Vanadium Dioxide (VO_2_)-Based Linear Optical Cavity Sensor

**DOI:** 10.3390/nano14040328

**Published:** 2024-02-07

**Authors:** Rana M. Armaghan Ayaz, Amin Balazadeh Koucheh, Kursat Sendur

**Affiliations:** 1 Faculty of Engineering and Natural Sciences, Sabanci University, 34956 Istanbul, Turkey; rayaz16@ku.edu.tr (R.M.A.A.); aminb@sabanciuniv.edu (A.B.K.); 2 Institute of Mechanical Intelligence, Scuola Superiore Sant’Anna, 56124 Pisa, Italy; 3 Center of Excellence for Functional Surfaces and Interfaces, Sabanci University, 34956 Istanbul, Turkey

**Keywords:** optical sensor, optical resonator, refractive index sensor, silicon-on-insulator (SOI) platform, vanadium dioxide, phase-changing materials, finite element method, resonant wavelength change, optical loss constant

## Abstract

Sensors fabricated by using a silicon-on-insulator (SOI) platform provide promising solutions to issues such as size, power consumption, wavelength-specific nature of end reflectors and difficulty to detect ternary mixture. To address these limitations, we proposed and investigated a broadband-thermally tunable vanadium dioxide (VO_2_)-based linear optical cavity sensor model using a finite element method. The proposed structure consists of a silicon wire waveguide on a silicon-on-insulator (SOI) platform terminated with phase-change vanadium oxide (VO_2_) on each side to provide light confinement. A smooth transmission modulation range of 0.8 (VO_2_ in the insulator state) and 0.03 (VO_2_ in the conductive phase state) in the 125 to 230 THz spectral region was obtained due to the of Fabry–Pérot (FP) effect. For the 3.84 μm cavity length, the presented sensor resulted in a sensitivity of 20.2 THz/RIU or 179.56 nm/RIU, which is approximately two orders of magnitude higher than its counterparts in the literature. The sensitivity of the 2D model showed direct relation with the length of the optical cavity. Moreover, the change in the resonating mode line width Δν of approximately 6.94 THz/RIU or 59.96 nm/RIU was also observed when the sensor was subjected to the change of the imaginary part *k* of complex refractive index (RI). This property of the sensor equips it for the sensing of aternary mixture without using any chemical surface modification. The proposed sensor haspotential applications in the areas of chemical industries, environmental monitoring and biomedical sensing.

## 1. Introduction

The use of optical fibers for the purpose of sensing has attracted increasing interest in recent years due to their immunity to electromagnetic interference, small size, low weight, and broad bandwidth [1,2,3]. Due to these attractive properties a number of experimental arrangements have been proposed that make use of optical fibers for monitoring various external parameters, including temperature, pressure, and vibration [4,5]. The absorption of light as it passes through the optical fiber surrounded by the analyte and the interaction between the evanescent field of optical fiber and material around it provides the basis of sensing. In order to increase this light-matter interaction side etching, polishing and tapering are among the most commonly used techniques [6,7,8]. These techniques to improve light-matter interaction for optical fibers are not only difficult and time consuming to implement but also sometimes harmful in nature because of the use of acid for etching the optical fiber. Moreover, these single-pass-type optical fiber sensors have limited sensitivity and resolution because light comes into contact only once with the analyte of interest and the effective path length (D_eff_) does not extend beyond few millimeters [9,10].

The emergence of micro-optical resonators and their use as a sensor marked the achievement of higher resolution and sensitivity. Some of these resonating structures, such as toroid [11] and micro-spheres [12], have a quality factor (Q-factor) in the range of 10^7^ to 10^8^. This indicates that light samples the analyte understudy many times before finally exiting the cavity and increases the effective path length significantly. However, the fabrication and corresponding coupling of light in these micro-optical resonators is not an easy task. External environmental factors, such as vibrations or thermo-optic effects, can significantly affect their measurements [13,14]. Although, linear optical resonators, such as those composed of two mirrors held parallel to each other and separated by a small distance, are able to address the aforementioned issues without affecting their performance. However, constraints such as the wavelength-specific nature of mirrors degrade their reflectivity with time, especially in corrosive environments, and proper alignment of reflectors for achieving a higher Q-factor restrict the application of linear optical resonators for the purpose of sensing. The use of a single-mode optical fiber (SMF) in combination with fiber Bragg gratings (FBGs) to act as a mirror on each end, leads to novel linear optical resonators [15,16]. Although fiber-optic-based linear resonators were able to solve mirror alignment issues and were suitable for use in the corrosive liquid environments, the wavelength-specific nature of FBGs remained a major issue. Similarly, most of the sensors discussed up to now are bench-top in nature, which means they require a much larger volume of specimen for analysis.

Due to advances in the SOI platform and its compatibility with complementary metal-oxide-semiconductor (CMOS) technology, the wavelength-scale optical elements are achieved [17,18]. A high refractive index contrast in SOI-based structures makes it possible to reduce the overall footprint of the sensor to just a few microns [19,20] by actively confining optical modes. Similarly, there is an increasing use of phase change materials (PCMs) in the integrated optical devices [21,22]. In addition to their relevance with CMOS fabrication techniques, they are also capable of broadband operations [23,24]. Compared to the other kinds of PCMs, vanadium dioxide (VO_2_) has successfully attracted the attention of many researchers. This is due to its ability to transition from an insulator monoclinic state to a metallic tetragonal rutile state at close to room temperature (68 °C) [25,26,27]. For VO_2_, this reversible insulator-to-metal phase transition is reached by applying the external agents that can be thermal, electrical and optical in nature [28,29,30]. Both of these two VO_2_ states greatly differ from each other in terms of electrical resistivity along with optical constants. From our previous work we know that at an operating wavelength of 1550 nm, the refractive index (RI) of VO_2_ showed a shift from 3.21 + 0.17*i* in the insulating phase to 2.15 + 2.79*i* while in the metallic phase [31].

It is a matter of great importance that, so far, the use of VO_2_ in combination with Si waveguide using SOI platform remained mainly limited to the fabrication of optical switches and modulators. Miller et al. developed non-resonant geometry and studied the optical switching behaviour of VO_2_ achieved by heating [32]. Again, by using Si and VO_2_, Arash Joushaghani et al. fabricated photodetectors in addition to switches based on the phenomenon of electroabsorption [33]. They reported a power level of less than 1 μW and 12 dB of extinction ratio (ER) with 5 dB as the insertion loss (IL). As a variation to Si, a Ge-based nanophotonics Fabry–Pérot (FP) resonator was fabricated to reach the milestone of 3 KHz as the modulation speed [34]. For this FP-type of resonating system, VO_2_ served as a fundamental element to achieve its broadband operation as well as tuning of reflection spectrum by means of applied electrical signal. In another study, the FP-cavity was used to enhance the absorption capacity in the visible and near-infrared regimes [35]. The design of the THz switchable filter with the FP-cavity resonator was achieved through the use of phase-changing material that is VO_2_ to establish an optical cavity [36]. Papari et al. developed a free space FP-type optically sensing structure by using switchable VO_2_ layers on a substrate as its end reflectors and reported its maximum sensitivity of about 0.87 THz/RIU [37]. Metamaterials are a relatively new breed of synthetic materials whose properties can be tailored as per user requirements and they were able to provide higher sensitivities [38,39,40]. Mehdi Aslinezhad theoretically and numerically investigated the semiconductor metamaterial in the terahertz band and obtained an RI sensitivity of 146,600 nm/RIU [41]. M. Askari et al. used commercial software package CST microwave studio, and a finite element frequency domain solver to design and simulate an infrared metamaterial-based RI sensor with a sensitivity of 2720 nm/RIU [42]. Khai Q. Le et al. applied electron beam lithography and the liftoff techniques to develop and use a nanostructured metal-insulator-metal metamaterial for label-free refractive index biosensing applications [43]. However, it is important to consider that the problems associated with the metamaterials such as inherent dissipation [44,45], narrowing the inter-nanostructure gap especially for metal lift-off fabrication methods [46,47], slow modulation and narrow tuning range that is not in the visible spectrum for the case of tunable metametrials [48] severely restrict their use for the optical sensing purpose.

In this manuscript, we proposed and simulated a silicon waveguide-based thermally tunable broadband FP optical cavity RI sensor. The presented resonating structure has a length of 3.84 μm and a width of 107 nm, and it demonstrated a sensitivity of about 20.2 THz/RIU for the real part of complex RI. The sensitivity for the real part of complex RI is almost 20 times higher than the one shown by Papari et al. [37]. Moreover, the proposed optical sensor is equally capable of sensing the change in the imaginary part of complex RI by means of varying the mode line width Δν. The recorded sensitivity for the imaginary part (optical loss constant) came out to be 6.94 THz/RIU. The measurement of Δν for the changing optical loss constant *k*, makes the sensitivity independent of the laser intensity fluctuations. This ability of the sensor to detect the change in each of the real and imaginary parts of complex RI showed its application for the concentration sensing of ternary mixture in addition to binary mixtures without using any chemical functionalization or surface immobilization technique, which is a difficult and time consuming process. Moreover, the use of VO_2_ in the presented model provided it the functionality to be used as an optical modulator. For the wavelength range of 125 to 230 THz, smoother modulation in transmission mode was attained between 0.8 (VO_2_ in the insulator state) and 0.03 (VO_2_ in the conductive phase state). This broadband linear optical cavity can be tuned to any wavelength by changing either its length or temperature of VO_2_ or both at the same time and eliminating the need for the wavelength-specific mirror. The presented linear optical cavity sensor finds its extensive applications in the fields of environmental monitoring [49,50], biomedical engineering [51,52], chemical and food industries [53,54].

## 2. Materials & Methods

**Figure 1:**
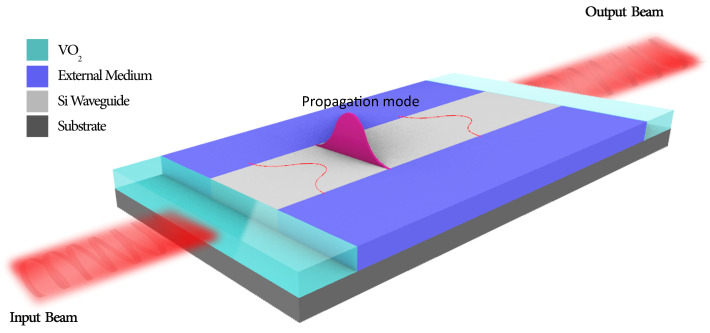
A 3D schematic of the proposed RI resonator.

**Figure 2:**
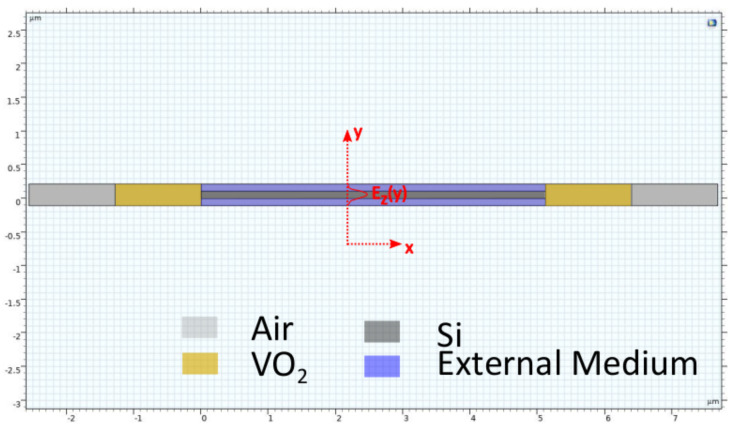
The proposed linear resonator model for RI sensing in COMSOL Multiphysics environment with the different colored regions defining Si waveguide, VO_2_, air and external medium. Similarly, the direction of light propagation is in the z-direction.

In this study, we propose a broadband-thermally tunable linear optical cavity sensor to address the limitations encountered in traditional optical sensors, such as wavelength-dependent nature of the mirrors, non-tunable nature, size and energy consumption. In Figure 1, a 3D schematic illustration of the proposed sensor is shown. In this model, VO_2_, which is shown with the turquoise blue, corresponds to the tunable region. The central region shows Si waveguide with 3.49 as its RI. On the top and bottom of the Si waveguide, an external medium serves as its cladding. For the design and simulation of the proposed linear optical resonator sensor, FEM-based COMSOL Multiphysics was used. As given in Figure 2, the proposed RI sensor model was studied and subsequently analyzed in the 2D environment of COMSOL. This environment was preferred because of the complexity of the proposed sensing structure, boundary conditions involved, associated time and RAM consumption for performing the analysis. Moreover, according to similar studies presented in the literature, performing the simulations in 2D or 3D in COMSOL has almost no effect on the final results. For the analysis, we used the Frequency Domain option provided in the Electromagnetic Waves, Frequency Domain (ewfd) of Wave Optics module. The geometric parameters used to design the model are given in Table 1.

“Free Triangular” mesh in “Predefined-Extremely Fine” settings with a minimum size element of 1.79 × 10^−4^ μm was used to resolve narrow regions. This meshing technique is preferred for 2D geometries because of its meshing speed, flexibility and adaptability to complex geometries. The sensor structure was excited at the range of frequencies spanning from 10 THz to 230 THz for observing any possible resonating modes.

The complex RI of the cladding is given by equation

(1) $$n_{\mathrm{comp}.}=n+ik$$

where *n* and *k* are the real and imaginary parts of it, respectively. The width of Si waveguide was chosen as 107 nm to ensure single mode operation [55,56]. For the purpose of absorbing any incident propagating wave or evanescent field, two perfectly matched layers (PML) were defined at the outer regions of the model. Similarly, at the top and bottom edges of the proposed model, a perfect magnetic conductor (PMC) boundary condition (BC) was applied. In the literature, the most common methods used to practically achieve the PMC condition are either by the application of high-impedance surfaces (HIS) [57,58,59] or by approximating perfect electronic conductors (PEC) to PMC by implementing modifications [60]. The scattering boundary condition (SBC) applied at the left and right sides makes these sides transparent only to incident plane waves. The combination of PML and SBC reduced any artificial reflection due to applied BCs.

For the case of the linear FP-cavity resonator of length *L_o_* having propagation constant β for its fundamental mode *LP*_01_ with *R*_1,2_ as the coefficients of reflection of its end reflectors, the transmitted power is given by

(2) $$T=\frac{\left(1-R_{1}\right)\left(1-R_{2}\right)}{{\left(1-\sqrt{R_{1}R_{2}}\right)}^{2}+4\sqrt{R_{1}R_{2}}sin^{2}[|\beta L_{o}+\frac{\varphi_{1}+\varphi_{2}}{2}\left|\right]}$$

The argument of sinus shown in Equation (2) should be a multiple of π, which is a necessary condition to observe the transmittance maxima while the FP-cavity is in the resonating condition [61,62]. For the speed of light *c* and considering *n_eff_* as the effective index of the cavity, the distance between two consecutive modes that is a free spectral range (FSR) can be found from expression [63,64].

(3) $$\Delta V_{\mathrm{FSR}}=\frac{c}{2n_{\mathrm{eff}}L}$$

However, for an applied frequency sweep range, only those modes will be supported by the cavity that will fulfil Equation (3). Here, it is important to mention that the Q-factor of the linear FP-cavity is directly linked to its length. For the N-number of round trips by light between two reflectors, the Q-factor of the linear resonator is given as in [65,66].

(4) $$Q=F\frac{2nL}{\lambda_{r}}$$

where λ_r_ is the resonant wavelength and the term *F = 2πN* is called the finesse of the cavity. By using relations given in Equations (1)–(4), the proposed linear resonator model was studied for its performance as an RI sensor. This is because any change in either the real, imaginary, or both parts of complex RI, as given by Equation (1), will result in a change in the resonant mode location Δλ_r_, its linewidth Δν_r_, or both phenomena occurring at the same time, respectively. The corresponding sensitivity in terms of resonant mode location change can be found by using Equation (5) [67,68], given below.

(5) $$S_{n}=\frac{\Delta \lambda_{r}}{\Delta n}$$

A similar kind of relation can also be defined for changes in mode linewidth as a function of variations in *k* [69,70,71]

(6) $$S_{k}=\frac{\Delta \nu_{r}}{\Delta k}$$

The anticipated fabrication steps start with the wafer preparation, which includes washing the wafer with acetone and drying it with a nitrogen blow gun. Next, the wafer is coated with HMDS (hexamethyldisilazane) to improve photoresist adhesion. In the next stage, wafer is coated with photoresist S1813 by using a spin coater to achieve the desired thickness of 1.4 μm. After that, it is baked at 115 °C for 60 s to evaporate unwanted solvent and improve the resist adhesion to wafer. The third step involves the transferring of the pattern from the mask to the substrate by using an aligner, e.g., EVG 620. The design on the mask stops the UV in such a manner that only the regions without a pattern are exposed to UV. In the development stage, areas exposed to UV are dissolved due to the positive nature of the photoresist used. Development is usually conducted using MF319 for 35 s, followed by hardbaking at 90 °C for 90 s to remove extra MF319 before etching. In the fifth or final stage, the etched wafer is placed in a vacuum chamber for pulsed layer deposition of VO_2_. Samples are heated at 500 °C. After that, ablation is conducted using vanadium metal as the target. Since the proposed model is based on the SOI platform, potential fabrication challenges can occur related to precise control of waveguide dimensions, proper mask alignment for pattern transferring, and coating material thickness, e.g., photoresist, deposition of VO_2_ layers on the structure, and coupling of light into the chip with the least possible insertion losses.

## 3. Results and Discussions

**Figure 3:**
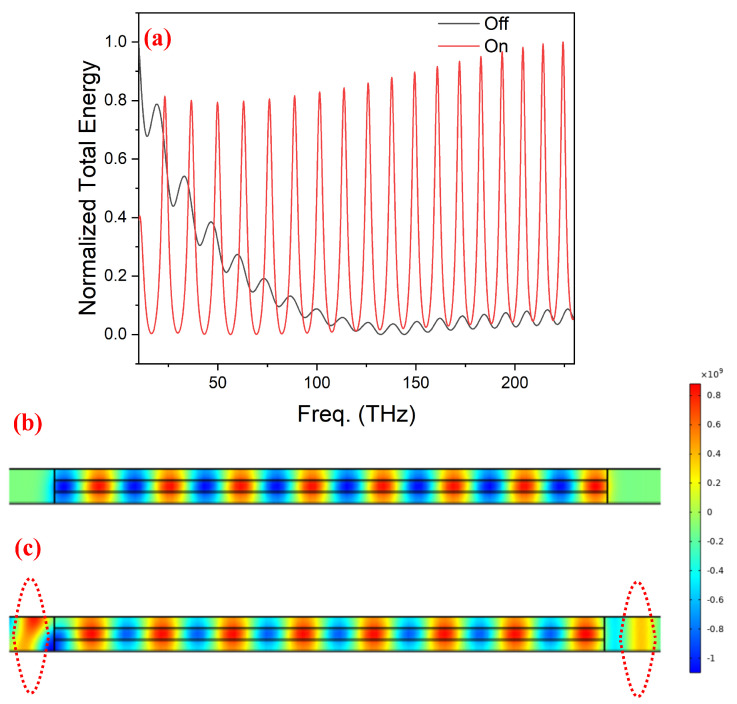
In the presence of air as the surrounding material of the sensor (**a**) plot of normalized energy density for the range of frequencies for the on (red) and off (black) states, which can be respectively translated as the conductive and insulator forms of VO_2_. Demonstration of modulation capability was made from the z-component of the surface electric field while VO_2_ in (**b**) the conductive phase and (**c**) the insulated phase, where at the location of dotted circles, leakage of the electric field can be seen.

We started with the working principle of a proposed brodaband-tunable RI sensor. This goal was achieved by examining the energy density inside the optical cavity at two different states of the VO_2_: insulator and metal. During this whole process of verification, air was considered as the surrounding medium. The results of the investigation have been shown in Figure 3 for the linear cavity having a length of 5.12 μm with 3.84 μm VO_2_ thickness. From Figure 3a, it can be seen that the tetragonal rutile metallic nature of VO_2_, which is opaque to input excitation, resulted in its trapping and led to the resonant cavity modes. On the other hand, the transparent nature of the insulator monoclinic VO_2_ resulted in the leakage of trapped energy and, hence, showed an exponentially decaying response. The presence of this leaked electrical field while VO_2_ was in insulator form can be seen in Figure 3c at the location of the red dotted circles, which istotally absent for the case of metallic VO_2_. The appearance of a strong evanescent field on both sides of the Si waveguide into the external medium confirmed the application of linear optical Si waveguide resonator as an RI sensor. Moreover, a closer observation reveals that with the help of the presented model, it is possible to achieve the transmission modulation between approximately 0.03 (insulated VO_2_) and 0.8 (metallic VO_2_) in the frequency range of 125 to 230 THz. Hence, making it a broadband optical modulator in addition to its potential application as an RI sensor [72].Transmission modulation was obtained because of the presence of the FP-phenomenon in the insulating phase of VO_2_ and its absence in its conductive phase. In the next step, for the purpose of optimizing the RI sensor model, resonating modes of the cavity were studied as the function of VO_2_ layer thickness on each side of the Si waveguide along with the length of the optical cavity resonator.

**Figure 4:**
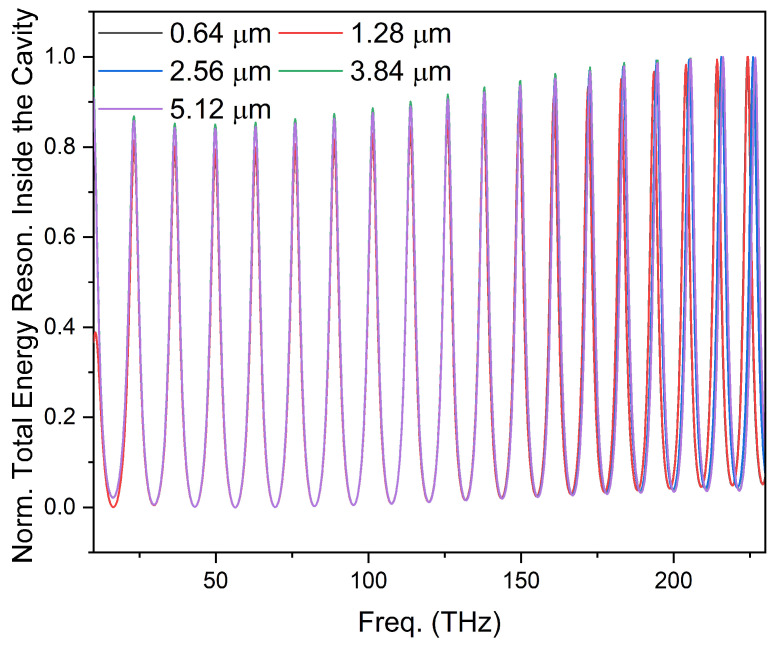
The resonating modes inside the linear optical cavity for the different thickness of VO_2_ layers. The resonance pattern remains almost same. Hence, the effect of increase in VO_2_ layer thickness can be neglected.

In the first scenario, the thickness of the VO_2_ layer was gradually varied from 0.64 μm to 5.12 μm and a corresponding effect on the resonating modes was investigated. The resulting mode pattern, as obtained for the different thickness of the VO_2_ layer, was plotted in Figure 4. As shown in Figure 4, it was observed that the increase in the thickness of the VO_2_ layer on each side of the Si waveguide has little or no effect on the resonating modes. Hence, its effect can be neglected. The light bounces back and forth between two VO_2_ layers inside the Si waveguide and encounters change in RI only once on each side of the waveguide, in contrast to the periodic variations of RI commonly observed in Bragg gratings or reflectors. For all the other analysis of our optical resonator RI sensor model, 1.28 μm was taken as the standard thickness of VO_2_ layer. The investigations were also made to study the detailed influence of the cavity’s length on resonating modes by considering FSR and Q-factor calculated from Equations (3) and (4), respectively, as standards.

**Figure 5:**
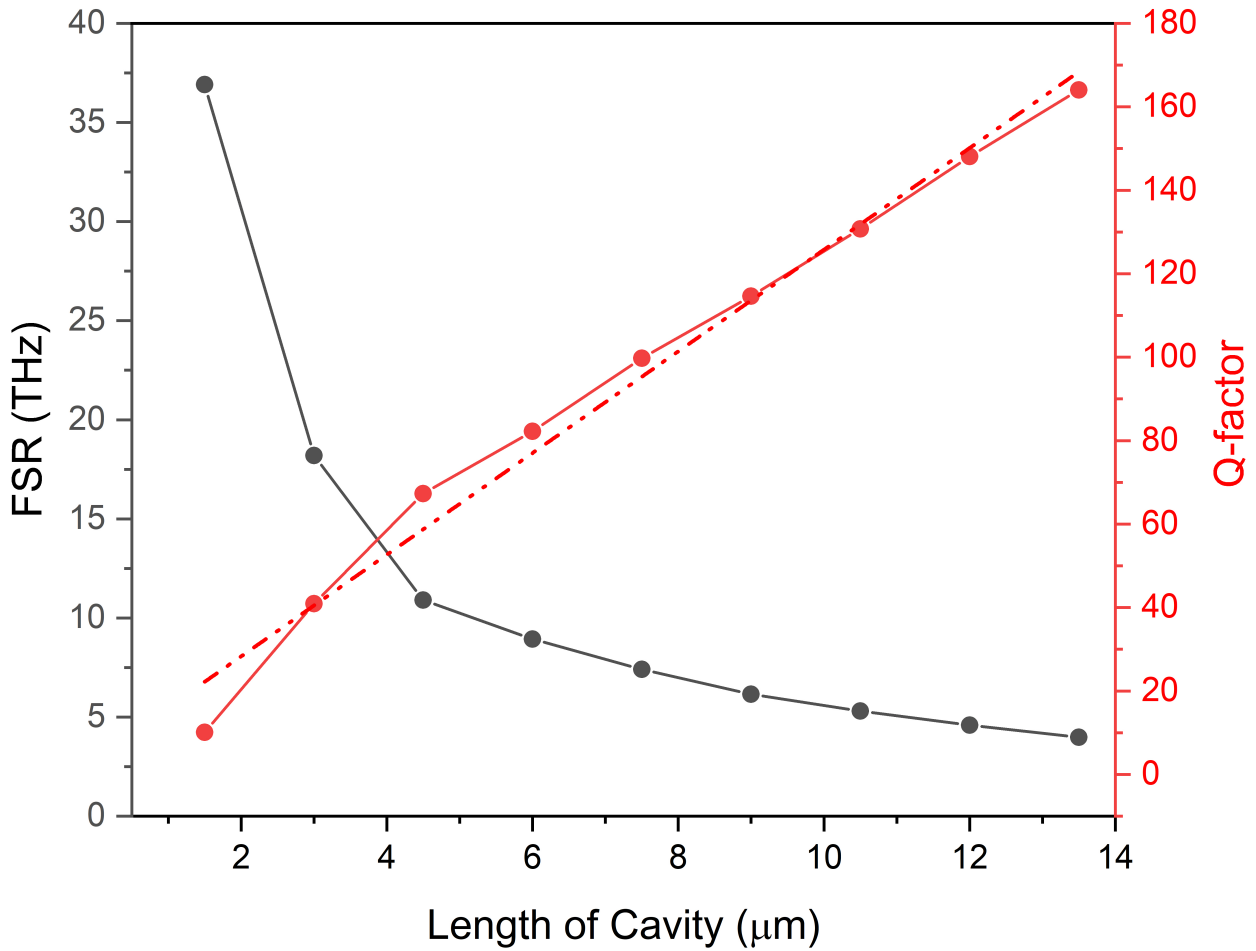
Change in free spectral range (black line) and Q-factor (red line) of the optical cavity as the function of its length. Q-factor curve along with its fitting (dotted red) shows an almost linear increase in it; however, there is also a reduction in FSR with the increase in cavity length.

The Q-factor serves as a figure of merit for any optical resonator and provides information about the losses of the cavity. An optical cavity with a lower loss will have a correspondingly higher Q-factor and the light will be able to make an increased number of round trips while sampling the analyte of interest each time before finally exiting the resonator. Both the higher number of round trips and the light-sample interaction translates itself in terms of higher sensitivity and resolution of the optical sensor. An optical resonator sensor with a high FSR has a well-differentiated resonance mode signal and can detect small changes in the concentration [73,74,75]. In the case of our proposed FP-type linear resonator-based RI sensor, as its length varies, resulting in an increase in Q-factor at the expense of reduced FSR, as governed by Equation (2). The relationship between Q-factor and FSR for different lengths of the cavity has been presented graphically in Figure 5. For the sake of achieving higher sensitivity with a reasonable FSR and Q-factor, the length of the linear optical resonator sensor was selected as 3.84 μm. This is a parameter given by the cross-point between FSR and Q-factor curve.

The linear optical resonator, optimized for its geometric parameters including cavity length, width, and thickness of VO_2_ layers, was then analyzed for its sensitivity to changes in refractive index (RI). To conduct this analysis, the real part *n* of the complex RI for regions (4) and (5), which represents the external medium in the model, was incrementally increased from 1.36 to 1.44.

**Figure 6:**
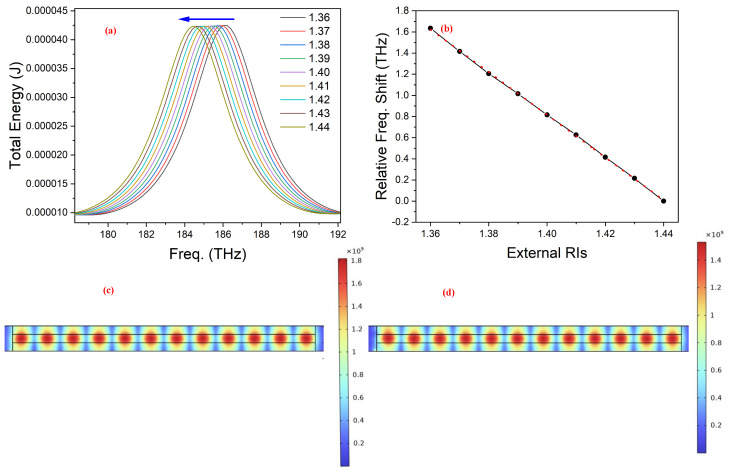
(**a**) The response of the 3.84 μm-long linear optical sensor to changing external RI. The arrow points to the direction of the shift in resonant mode location as the real part *n* of the complex external RI is changed. In (**b**), a plot of the relative frequency shift for the corresponding outside RI change (black line) with the slope of the linear fitting (dotted red line) providing the sensitivity of the proposed RI sensor. Surface electric field norm at 186 THz when external RI were (**c**) 1.36 and (**d**) 1.44, respectively.

**Figure 7:**
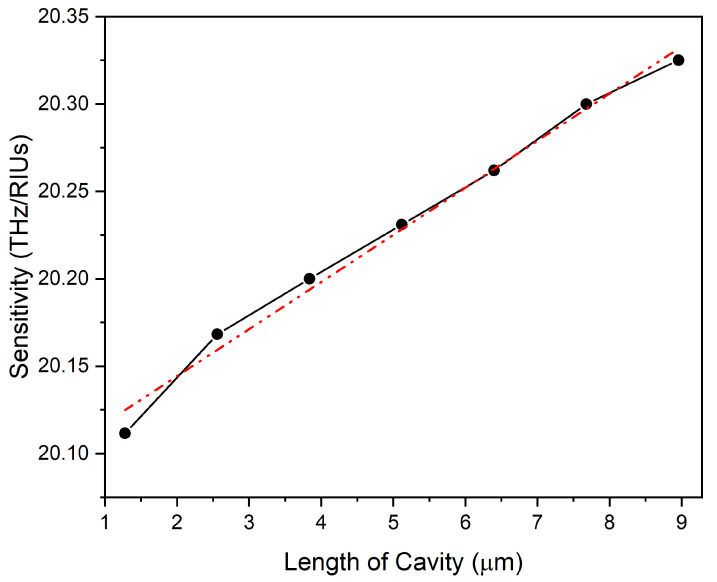
Linear dependence of the sensitivity for the proposed linear optical RI sensor on its length marked by the black line and fitted with the straight red line curve (dotted).

From Equation (2), the change in external RI of the medium surrounding the optical resonator manifests itself in the form of an increase in effective RI of the resonating modes, and hence, a change in their position. As can be seen in Figure 6, for the presented linear FP-type RI sensor with a length of about 3.84 μm, we observed the blue shift in the frequency spectrum for the increase in the real part of the external RI. The slope of the linear fit for the total shift in resonant mode location for the whole change of external RI provided the sensitivity. The recorded sensitivity for the real part of the RI sensor was 20.2 THz/RIUs or 179.56 nm/RIU. This sensitivity value is almost 20 times higher than the one reported by the G.P Papari et al. [37]. In the literature, G.P Papari et al. [37] presented an experimental study on an optical sensor system. The experimental and analytical results in that study show similar trends to our work, which include switching (conductor–insulator) behaviour of VO_2_, change in FSR and Q-factor of the cavity with changes in the cavity’s length, and change in resonant peak location with a change of the external RI. These findings support the validity of the computational work on modelling of our sensor. This recorded increase in sensitivity can be attributed to the following: (i) the use of dielectric Si waveguide to reduce the FWHM of the resonating modes by mitigating the optical cavity losses that are caused by issues such as attenuation or scattering, as in the case of air; (ii) proper guiding of light in the Si-based aligned optical cavity instead of the free space optical cavity where a minor misalignment of VO_2_ end reflectors can cause enhanced optical losses affecting Q-factor (iii) Improved Q-factor and longer FSR, respectively, due to lesser intrinsic losses and reduced length of the linear optical cavity resonator. Similarly, a decrease in the surface electric field norm and an increase in the evanescent field was recorded for the rising external RI, as shown in Figure 6c,d. This, in turn, leads to stronger light–matter interaction. The sensitivity of the reported optical resonator RI sensing model displayed the direct relation with its length as shown in Figure 7. This is because of the increase in Q-factor with cavity length, as presented in Figure 5, which enabled the light to sample the analyte of interest many times before finally exiting the optical resonator.

**Figure 8:**
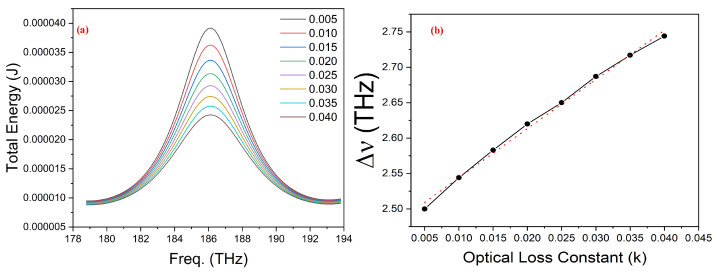
(**a**) Change in line width (Δν) of resonating mode with the variation of optical loss constant *k* (**b**) Plot of line widths (Δν) change obtained by applying Lorentz fit for different optical loss constants. The slope of the linear fit (dotted red line) on the real data line (black) provides the sensitivity of the sensor to an optical loss constant.

The concentration of mixtures with a single unknown parameter, such as binary mixtures, can be sensed by optical sensors capable of detecting changes in the real part of the refractive index (RI). But for the case of two unknown parameters as they apply to ternary mixtures, this technique is not useful anymore. For determining the concentration of such mixtures, a more complex and sophisticated method such as functional group immobilization is used. This, in turn, renders the optical sensing system less sensitive and adds to its final cost under the fold of materials used for the surface functionalization of the sensor [76,77]. In the literature, it is established that each of the individual components making up a ternary mixture, having the same real part, has different imaginary parts [78,79].This basic principle was used to test the ability of the proposed broadband optical sensor to detect ternary mixtures. While keeping the real part *n* of the complex external RI fixed at 1.36, the optical loss constant *k*, or imaginary part of it, was changed in equal steps from 0.035 to 0.01. The linewidth of the resonating cavity mode Δν at FWHM, calculated by applying Lorentzian fitting, showed an increase. The increase in Δν can be attributed to an increase in cavity losses with the increase in the optical loss constant *k* for the surroundings of the sensor. Figure 8a shows the increase in Δν of the mode for different values of the optical loss constant *k*. The plot in Figure 8b, which shows the change in FWHM of the resonating mode found from Lorentz fitting against *k*, demonstrates that the increase in Δν is almost linear. This provides an imaginary part sensitivity of 6.94 THz/RIUs, which is equivalent to 59.96 nm/RIU. Therefore, the reported sensor is equally capable of detecting ternary mixtures without the need for any surface immobilization methodology. The sensitivity comparison of our modeled sensor with the devices reported in the literature can be seen in Table 2.

## 4. Conclusions

In conclusion, a broadband-thermally tunable linear optical resonator based on a silicon wire waveguide coated with vanadium oxide was proposed and computationally demonstrated using COMSOL Multiphysics. The reported optical resonator achieved transmission modulation between 0.8 and 0.03 in the frequency range of 125 to 230 THz for VO_2_ in insulator and conductive phases, respectively. This modulation behavior of the model can be attributed to the Fabry–Perot phenomenon. The presented linear cavity model demonstrated a sensitivity of about 20.2 THz/RIU or 179.56 nm/RIU to the real part of the complex refractive index. This sensitivity value is more than an order of magnitude higher than those reported in the literature. The improvement in sensitivity can be mainly attributed to the use of a Si waveguide with lower optical losses, the application of VO_2_ as end mirrors for the Si waveguide to eliminate misalignment issues, and the enhanced quality factor due to reduced cavity losses.

For the reported RI sensing model, a direct dependence of sensitivity on resonator length was also observed. Moreover, the change in the linewidth of the resonating mode for the linear cavity sensor as a function of the change in the optical loss constant *k* made it equally useful for sensing ternary mixtures in addition to binary ones. The achieved sensitivity for the reported linear cavity sensor model in the case of optical loss constant *k* was obtained as 6.94 THz/RIU (59.96 nm/RIU). The mode linewidth measurements for variable *k* make the resulting sensitivity immune to laser intensity fluctuations. The sensor design and the findings of this study can be applied in various areas, including chemical engineering, biological sciences, food industries, and environmental monitoring.

## Figures and Tables

**Table 1:** Geometric parameters used for the different layers.

Name of Layer	Parameter Name	Value (nm)
Si	Width (W_Si_)	107
	Height (H_Si_)	W_Si_/2
	Length (L_Si_)	variable
SiO_2_	Height (H_SiO_2__)	2000
	Length (L_SiO_2__)	variable

**Table 2:** Comparison of the proposed RI sensor with others found in the literature.

Sensing Arrangements	Sens. (nm/RIU)	Oper. Range (RIU)	Ref.
FBG and spherical-shape structure	2.87	1.357–1.458	[80]
Optical fiber Mach–Zehnder	57.62	1.35–1.40	[81]
Fiber tip integrated whispering gallery mode mirco-ring resonator	63	1–1.33	[82]
Ge–Sb–Se chalcogenide microring resonator	123	1.3328–1.342	[83]
FDTD and 2D FEM analysis of ring resonator	146	1.35–1.39	[84]
VO_2_ based Optical Resonator	179.56	1.36–1.44	This study

## Data Availability

The data that support the findings of this study are available from the corresponding author upon reasonable request.
